# Comparing Infection Profiles of Expectant Mothers with COVID-19 and Impacts on Maternal and Perinatal Outcomes between the First Two Waves of the Pandemic

**DOI:** 10.3390/jpm11070599

**Published:** 2021-06-25

**Authors:** Yolanda Cuñarro-López, Pilar Pintado-Recarte, Concepción Hernández-Martín, Pilar Paya-Martínez, Rocío López-Pérez, Ignacio Cueto-Hernández, Javier Ruiz-Labarta, Óscar Cano-Valderrama, Óscar Martínez-Pérez, Coral Bravo-Arribas, Miguel A. Ortega, Juan Antonio De León-Luis

**Affiliations:** 1 Department of Public and Maternal and Child Health, School of Medicine, Complutense University of Madrid, 28040 Madrid, Spain; ycunarro@ucm.es (Y.C.-L.); pintadorec@yahoo.es (P.P.-R.); chermartin@telefonica.net (C.H.-M.); mpilar.paya@salud.madrid.org (P.P.-M.); ignaciocuetohernandez@gmail.com (I.C.-H.); javruila@hotmail.com (J.R.-L.); cbravoarribas@gmail.com (C.B.-A.); jaleon@ucm.es (J.A.D.L.-L.); 2 Department of Obstetrics and Gynecology, University Hospital Gregorio Marañón, 28009 Madrid, Spain; 3 Health Research Institute Gregorio Marañón, 28009 Madrid, Spain; oscarcanovalderrama@hotmail.com; 4 Department of Obstetrics and Gynecology, Hospital Universitario Santa Lucia, 30202 Cartagena, Spain; rocio.lopez.perez@gmail.com; 5 Obstetrics and Gynaecology Department, Puerta de Hierro University Hospital, 28222 Madrid, Spain; 6 Department of Medicine and Medical Specialities, Faculty of Medicine and Health Sciences, University of Alcalá, 28801 Alcalá de Henares, Spain; miguel.angel.ortega92@gmail.com; 7 Ramón y Cajal Institute of Sanitary Research (IRYCIS), 28034 Madrid, Spain; 8 Cancer Registry and Pathology Department, Hospital Universitario Principe de Asturias, 28806 Madrid, Spain

**Keywords:** COVID-19, pregnancy, maternal and perinatal medicine, morbidity

## Abstract

During 2020, Coronavirus Disease-19 (COVID-19) incidence fluctuated in two clear waves across the spring and autumn periods. This study was designed to compare the maternal and perinatal clinical outcomes in obstetrics patients with COVID-19 between the two waves of infection in Spain. We conducted an observational, analytical, ambispective cohort study with longitudinal follow-up of mothers with confirmed SARV-CoV-2 infection from different hospitals in our country between March–November 2020. We recruited 1295 pregnant women with SARS-CoV2 infection from 78 hospitals, 846 (65.3%) of whom were diagnosed during the first wave and 449 (34.7%) during the second wave. Our results show that patients developing COVID-19 during the first wave had more symptoms at triage, early in pregnancy with greater rates of COVID-19-related maternal morbidity; caesarean section and preterm birth in the first wave. We register two cases of maternal mortality and only during the first wave. Maternal morbidity events showed a strong link to perinatal mortality events in the first wave compared to the second wave, in which maternal morbidity was more associated with pneumonia. Likewise, maternal morbidity showed a strong correlation with perinatal morbidity events in both waves. We describe the differences between the patients’ profiles and management between the two waves and related to maternal and perinatal outcomes. Differences were also observed in the management of pregnant women with COVID-19. Thus, there were fewer caesarean sections, and maternal and perinatal morbidity events were reduced in the second wave, while the impacts of respiratory symptoms and their severity, including a greater need for maternal treatment, were greater in this last period. Identifying the impact that changes in the profile as well as in the treatment have on maternal–perinatal morbidity and mortality will help improve the well-being of our patients and their newborns.

## 1. Introduction

The emergence and rapid global spread of severe acute respiratory syndrome coronavirus 2 (SARS-CoV-2) causing coronavirus disease 2019 (COVID-19) is responsible for the pandemic in which we are now immersed. As of 31 March 2021, over 128,000,000 confirmed cases of COVID-19 and 2,797,000 associated deaths worldwide have been reported. Spain is one of the most severely affected European countries after Russia, France or the UK, with its 3,279,825 confirmed cases, including 75,199 deaths so far [1].

Physiologic or immunologic changes during pregnancy may have systemic effects that predispose women to complications from respiratory infections that lead to maternal and fetal mortality and morbidity [2,3]. However, the clinical characteristics of pregnant women with COVID-19 seem to resemble those of non-pregnant women, and most expectant mothers with SARS-CoV-2 infection show only mild or no symptoms [4]. Thus, maternal and perinatal outcomes seem favorable, especially in women who are asymptomatic or show a milder presentation [5]. Notwithstanding, studies have shown that pregnancy in itself may be a risk factor for death, pneumonia and ICU admission in SARS-CoV-2-infected women of reproductive age [6]. Further, a major risk factor for an adverse outcome in patients with COVID-19 is the presence of comorbidities, including advanced age [7], diabetes, hypertension and obesity [8].

In our country, as in many others, the incidence of SARS-CoV-2 has fluctuated throughout the pandemic and two clear waves were differentiated over the year 2020, the first between March and June 2020, and the second showing a more gradual rise from the summer months, remaining practically stable until the start of December [9]. During the first wave, many people with symptoms of COVID-19 in Spain did not have a RT-PCR test including obstetrics patients [10] and the general population [11]. In the second wave, the clinical severity of SARS-CoV-2 infections seemed significantly reduced, most likely reflecting a lower viral burden as a result of social distancing, increased use of face masks, promotion of outdoor activities and restrictions on gatherings [12] reflecting more detected asymptomatic cases in the population at large.

To address this possible changing pattern of infection, the present study was designed to compare between the two waves maternal, obstetric and perinatal characteristics along with maternal–perinatal mortality–morbidity events in obstetrics patients with COVID-19 in Spain.

## 2. Materials and Methods

### 2.1. Study Design

This was an observational, analytical, ambispective cohort study with longitudinal follow-up performed according to Strengthening the Reporting of Observational Studies in Epidemiology (STROBE) guidelines [13].

Participants were expectant mothers registered by the Spanish Obstetrics Emergency group, recruited from several Spanish public and private centers. This national registry was started in March 2020 to determine the morbidity caused by COVID-19 in the mother–baby binomial, and to monitor the interventions and measures necessary to improve the care given to these patients [14].

Data were collected using a standard form and entered by the lead researcher at each center after child birth and over a follow-up of six weeks postpartum. All the patients were assessed six weeks after delivery; however, not all the patients had all the variables evaluated at this time.

SARS-CoV2 infection was confirmed by detecting viral RNA by real-time reverse transcription polymerase chain reaction (RT-PCR) conducted on a nasal swab, sputum or throat swab sample [10,15].

Inclusion criteria were obstetrics patients diagnosed with COVID-19 during their pregnancy and parturition. Patients were excluded if follow-up could not be completed over the 6 weeks after delivery.

The study period ran from 1 March 2020 to 3 November 2020. The two waves of the pandemic in Spain were defined according to the data published by the Centre for Coordination of Health Warnings and Emergencies of the Spanish Ministry of Health (REF). Accordingly, all cases produced between 1 March 2020 and 30 June 2020 were assigned to the first wave. During this wave, 249,271 cases and 28,355 deaths due to COVID-19 were registered in Spain [16]. For the second wave, patients included were those diagnosed with SARS-CoV-2 infection between 1 July 2020 and 3 November 2020, which was the last recruitment date. During this latter period, 1,010,095 cases and 8140 deaths due to COVID-19 were registered [17].

The data compiled were: date of diagnosis; number of obstetrics patients with active infection in each wave; maternal characteristics including age, tobacco use, body mass index (BMI) (kg/m^2^), morbidities (including diabetes, hypertension and others), nulliparous/multiparous, COVID-19 symptoms at triage, presence of pneumonia and need for treatment; maternal laboratory findings including complete blood count and coagulation parameters such as hemoglobin, hematocrit and leucocyte count, lymphocytopenia (lymphocyte count less than 1500 cells per cubic millimeter), thrombocytopenia (platelet count less than 150,000 per cubic millimeter) and activated partial thromboplastin time (aPTT); obstetric and perinatal characteristics including obstetric morbidities, gestational age (GA) at triage, GA at delivery and neonate birth weight; maternal and perinatal mortality; maternal morbidity including both COVID-19-related morbidity (defined as need for oxygen therapy if oxygen saturation in room air at rest was <94%, need for mechanical ventilation and maternal admission to an Intensive Care unit (ICU)) and non-COVID-19-related morbidity such as C-section, hemorrhagic maternal disorders (abruptio placentae, accreta/increta/percreta placenta, postpartum hemorrhage or ruptured uterus) and hypertensive disorders (hypertension, preeclampsia, eclampsia, hypertensive encephalopathy or HELLP syndrome) [18]; and perinatal morbidity including preterm birth (GA at birth less than 37 weeks), Apgar score at 5 min < 7 and admission to the Neonatal Intensive Care unit (NICU).

### 2.2. Data Analysis

Data compiled for the study were entered in a Microsoft Office Excel database, version 16.42 (Microsoft, Redmond, WA, USA) and statistical analysis of data was performed using the package Stata 13.1 (StataCorp LLC, College Station, TX, USA).

An analytical study was conducted comparing the main maternal characteristics, laboratory test results, obstetric and perinatal characteristic and maternal/neonatal mortality–morbidity data between the two waves. Significance was set at *p* < 0.05. Quantitative variables were expressed as the mean (interquartile range or 95% confidence interval (CI)) and categorical variables as number of patients and rates (%) (95% CI). Univariate analysis was performed using Fisher’s exact test, chi-squared test or Student’s *t*-test, as appropriate.

Next, we compared between the two waves the relationship between maternal and perinatal morbidity and, through multivariate logistic regression analysis, the maternal, obstetrics and perinatal data collected. Variables proving significantly different or clinically meaningful in the univariate analysis (odds ratio (OR) > 1.5, OR < 0.67, or Pearson correlation coefficient > 0.1) were entered in the multivariate analysis. Finally, ORs and 95% CIs obtained in the multivariate analysis were graphically represented for maternal, obstetric and perinatal variables. Those proving significantly different and/or clinically relevant more related with maternal-neonatal morbidity in each of the two waves are provided on a scale of grey.

### 2.3. Ethical Approval

The authors declare no conflict of interest. All procedures involving human participants were in accordance with the ethical standards of the institutional and/or national research committee and with the 1964 Helsinki declaration and its later amendments or comparable ethical standards. The registry’s objective updates were approved by the coordinating hospital’s Medical Ethics Committee (Ref: PI 55/20) and each collaborating center subsequently obtained protocol approval locally. The registry protocol is available at ClinicalTrials.gov, identifier NCT04558996. For inclusion in the registry, patients were required to sign an informed consent form. In cases when this could not be obtained because of the emergency of the care given or because of insufficient individual protective equipment available, consent was verbal and noted as such in the patient’s medical record.

## 3. Results

**Figure 1:**
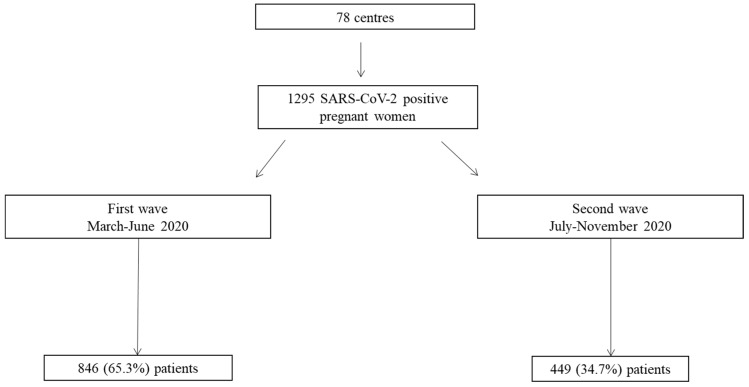
Flowchart of patients recruited during the first and second waves of the pandemic.

Over the period 1 March to 3 November 2020, we recruited 1295 pregnant women with SARS-CoV2 infection from 78 hospitals, 846 (65.3%) of whom were diagnosed during the first wave and 449 (34.7%) during the second wave (Figure 1). All centers reported their data for the first wave and 63 (80.8%) also did so for the second wave. Taking as reference the number of cases of COVID-19 diagnosed and the number of RT-PCR test performed in each of the two periods reported by the Spanish Ministry of Health [16,17], the rate of positive RT-PCR test was 6.8% in the first wave and 9.9% in the second wave. The pregnant women in our cohort accounted for 33.9 of every 10,000 infections in the first wave, and 4.4 of every 10,000 infections in the second wave.

Table 1 shows the maternal characteristics, laboratory findings, obstetrics and perinatal data and maternal/neonatal mortality and morbidity recorded in each wave of the pandemic. As may be noted, patients developing COVID-19 during the first wave showed an older mean age, more symptoms at triage along with higher rates of pneumonia and leukocytosis and lymphopenia. In addition, GA at diagnosis was younger, COVID-19-related maternal morbidity was greater and there was a higher rate of caesarean section and preterm birth in the first wave (all *p* < 0.05). No differences were detected in the remaining variables examined. Fetal mortality rate, which is included in the perinatal mortality rate, was also similar in both periods (0.5% vs. 0.5% in the first and second wave, respectively, *p* = 0.889).

All the patients were assessed six weeks after delivery; however, information about some variables (such as hemorrhagic and hypertensive disorders) were not available for all the patients.

Table 2 describes the univariate analysis performed comparing maternal and neonatal morbidity and the maternal, obstetric and perinatal variables examined in each of the waves. Overall, it may be observed that some 40% of the patients of both waves experienced maternal morbidity events, while neonatal morbidity was slightly greater in patients of the first wave (20.4% vs. 16.6%).

However, perinatal morbidity was linked to a 5 times greater likelihood of maternal ICU admission and a maternal hypertensive disorder in pregnant women with COVID-19 in the second wave compared to those diagnosed in the first wave (OR: 52.9 vs. 11.6 and OR 23.5 vs. 4.3, respectively) while the need for maternal mechanical ventilation was associated with a 10-fold greater probability of perinatal morbidity in mothers in the first wave compared to the second wave (OR: 10.4 vs. 1.0). Other variables related to perinatal morbidity in the first wave were maternal morbidities (OR: 1.7), symptoms at triage (OR: 1.9), obstetric morbidities (OR: 2.1), GA at delivery (r: −0.7), birthweight (r: −0.5), overall maternal morbidity (OR: 3.8), COVID-19 maternal morbidity (OR: 3) and not COVID-19 maternal morbidity (OR: 3.9). In the second wave, obstetric morbidities (OR: 4.6), GA at triage (r: −0.4), GA at delivery (r: −0.6), birthweight (r: −0.4), overall maternal morbidity (OR: 3.3), COVID-19 maternal morbidity (OR: 10.4) and not COVID-19 maternal morbidity (OR: 3.4) were also linked to perinatal morbidity.

**Figure 2:**
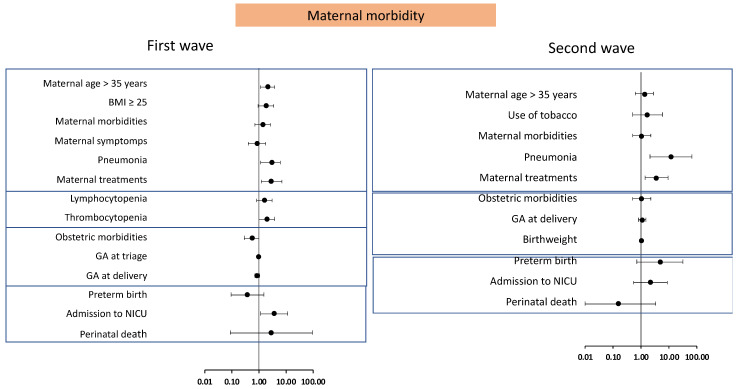
Forest plot of odd ratios and 95% confidence intervals of correlations in maternal and perinatal variables versus maternal morbidity in the first and second waves of the pandemic obtained by multivariable logistic regression.

Figure 2 shows the relationship detected in the multivariate analysis between the maternal, obstetric and perinatal variables emerging as significant in the univariate analysis and overall maternal morbidity in each of the two waves. The variables significantly linked to the morbidity of the pregnant women in the first wave were age older than 35 years (OR 2.1, 95% CI 1.1–3.8), need for treatment (OR 2.8, 95% CI 1.3–6.3), thrombocytopenia (OR 2.0, CI 95%: 1.0–3.8) and ICU admission (OR 3.7, 95% CI 1.2–11.8). In contrast, those associated more with the morbidity of patients in the second wave were the presence of pneumonia (OR 11.6, 95% CI 2.0–65.8) followed by a need for treatment (OR 3.3, CI 95%: 1.3–8.4). Maternal age older than 35 years, smoking, premature birth and a need for NICU admission were also positively associated with maternal morbidity in the second wave although the difference lacked significance.

**Figure 3:**
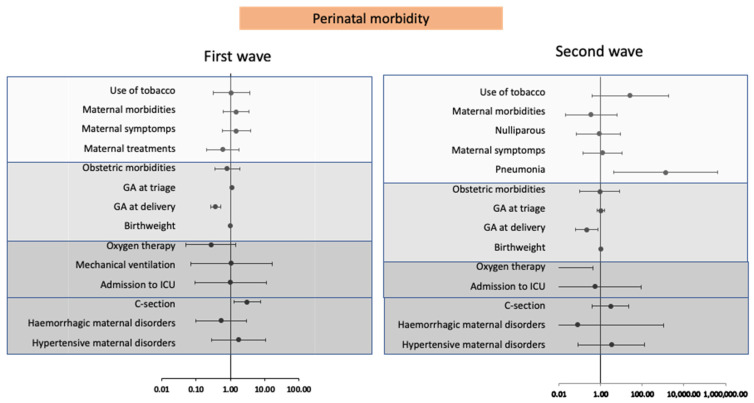
Forest plot of odd ratios and 95% confidence intervals of correlations in maternal and perinatal variables versus perinatal morbidity in the first and second waves of the pandemic obtained by multivariable logistic regression.

Similarly, our multivariate model in Figure 3 shows that C-section deliveries in the first wave were significantly related to a greater likelihood of perinatal morbidity (OR 3.1, 95% CI 1.3–7.5). Further, in this period, patients with comorbidities and symptoms suggestive of COVID-19 and also undergoing hypertensive morbidity events showed a greater likelihood of morbidity of their newborns although without significance. In contrast, in the second wave, maternal pneumonia was the variable mostly associated with events of perinatal morbidity (OR 1301.2 CI 95%: 4.1–414,584.7), while smoking, C-section delivery and hypertensive morbidity in the mother were positively correlated with neonatal morbidity, but again without observing significant differences.

## 4. Discussion

This study was performed on 1295 pregnant women recruited from 78 centers across Spain with active SARS-CoV-2 infection confirmed during the first (*n* = 846, 65.3%) and second (*n* = 449, 34.7%) waves of the pandemic in 2020. Significant differences were detected between the two time periods in clinical variables in that patients in the first wave were older and showed more symptoms suggestive of COVID-19 at triage, a greater presence of pneumonia and also more analytical abnormalities such as leukocytosis and lymphopenia. Further, during the first wave, infection diagnosis in the mother occurred earlier in pregnancy, maternal COVID-19-related morbidity events were more frequent, and greater proportions of C-sections and premature births were recorded. Our multivariate analysis designed to explain maternal and perinatal morbidity revealed different profiles of the first and second waves in terms of both values of the variables examined and their impacts.

This study provides data for the larger series reported to date, along with the national cohort described by Martínez-Portilla R.J. et al. [6] including 5182 pregnant women from 475 hospitals in Mexico, the study by Mullins E. et al. [19] including 4005 pregnant women from the UK and USA, or the multinational cohort described by D’Antonio F. et al. [20] including 887 pregnant women from 25 countries. The difference observed in the incidence of SARS-CoV-2 infection in expectant mothers in the first two waves of the pandemic in Spain (33.9 vs. 4.4 per 10,000 SARS-CoV-2 infections, respectively) may be explained by underdiagnosis and underreporting during the first wave of COVID-19 in Spain, as proposed by Pollán M. et al. [11] in their Spanish National Seroepidemiological Study conducted from 27 April to 11 May 2020. These authors claimed that the real number of infections in the first wave was about 10 times higher than the cases confirmed. During this period, many people with symptoms compatible with COVID-19 did not have a PCR test [10] and at least one-third of infections determined by serology were asymptomatic [12]. Further, as from the first weeks after the start of the pandemic, many Spanish maternity units screened all asymptomatic pregnant women admitted because of any obstetric indication for SARS-CoV-2 [14], thus favoring a positive diagnosis in the obstetric population. In parallel, the general asymptomatic population or those with mild symptoms have been underdiagnosed as these individuals did not visit their health center and remained at home because of the severe mobility restrictions and lockdowns imposed. From March to June 2020, there were 111,095 births in Spain. In the same period of time in 2019, 120,206 births were registered [21]. Therefore, there was a 7.6% reduction in the birth rate during the first wave of the COVID-19 pandemic. Although there are no data about the birth rate in the second semester of 2020, a higher reduction is expected in this period. Uncertainty about the effect of SARS-CoV-2 infection on maternal and perinatal outcomes could be one of the reasons because women, mostly if they had a high obstetric risk, could have delayed the pregnancy. Moreover, the economic crisis and the lockdown of the assisted reproduction services could also have helped to this reduction during the second wave of the pandemic.

Table 1 reveals that the older age of patients in the first wave, as reported by others [22], along with the presence of comorbidities such as diabetes or chronic hypertension [23], or coexisting obstetric morbidities such as preeclampsia [24], are risk factors for the development of more severe COVID-19 [20,25]. An increased level of maternal COVID-19 symptoms, reflected by the pneumonia rate or the need for oxygen therapy and mechanical ventilation during the first months of the pandemic, has been reported previously [26,27]. Besides, it has also been highlighted that pregnant women inherently have increased odds of death, pneumonia and ICU admission compared to non-pregnant women [6]. Further, in the first few months, a lack of knowledge of the impacts of the infection on the mother–baby binomial along with quick respiratory worsening of the expectant mother requiring urgent early termination of pregnancy gave rise to an increase observed in caesarean deliveries and therefore preterm births in the first wave [10,27]. Our study did not observe any significant difference in the maternal–perinatal mortality or in the need for ICU admission of the mother or neonate between the two waves. Although we did not find any differences in the fetal mortality, it could be possible that maternal infection could be related with an increased in the fetal death rate. Therefore, the fetuses with maternal infection have a lower likelihood of survival to birth and the birth cohort may only represent infants and mothers with less severe infection symptoms as well as lower risks for maternal and perinatal outcomes.

It seems that cases of active COVID-19 in pregnant women during the second wave were clinically less severe than during the first wave, as described by Soriano V. et al. in their study performed on the non-pregnant adult population of Madrid [12]. During the second wave, the extended use of masks, social distancing and restrictions on mobility, gatherings, etc., could have reduced the viral burden, which is thought to be proportionally related to the risk of showing more severe disease [28]. Villalaín C. et al. [5] in a seroprevalence analysis of pregnant women living in south Madrid, which is one of the Spanish cities showing a higher incidence of SARS-CoV-2 infection [17], detected a rate of up to 21.3% seropositivity for SARS-CoV-2. We propose several factors leading to less severe cases of COVID-19 in our patients in the second wave, implying a lower risk of maternal–perinatal morbidity events. These include reduced exposure to the virus, along with an improved diagnostic capacity to detect cases in patients with null or mild symptoms allowing for their earlier identification and isolation, and acquired knowledge about the pathophysiology and management of COVID-19 such as a younger average age of infected individuals and the benefits of the earlier use of corticosteroids [12].

Since the outbreak of the pandemic, several studies have examined pregnancy as a potential risk factor for morbidity–mortality events [6]. Consistently, it can be observed in Table 2 that practically one in every two of our patients with active SARS-CoV-2 infection in both waves experienced maternal morbidity events. A possible cause is that SARS-CoV-2 enters the cell via the angiotensin-converting enzyme 2 (ACE2) receptor, which is upregulated in normal pregnancy as a result of higher ACE2 expression [29]. Besides, physiological changes in the immune and cardiopulmonary systems during pregnancy mean that pregnant women may be more susceptible to developing more severe symptoms after infection with a respiratory virus [30]. Knowledge acquired about infection management and the different behavior of the pandemic as it unfolded, could explain the differences observed here in neonatal morbidity events that were more frequent in the first wave (20.4% vs. 16.6%), premature birth being the main adverse perinatal outcome [31]. Moreover, owing to the COVID-19 mitigation measures implemented, it is likely that the decline in the overall incidence of preterm birth could be attributable to one of its main risk factors, i.e., asymptomatic maternal infection, which through vertical transmission can cause intrauterine infection, initiating a cascade resulting in preterm birth [32]. Physical distancing and self-isolation, lack of commuting, closing of schools and childcare facilities, promoting outdoor activities, an increased awareness of hygiene (e.g., hand washing), a substantial reduction in air pollution [33], the closure of non-essential businesses and obligatory home assignments probably resulted in less physically demanding work, reduced work-related stress, more hours of sleep, exercise indoors and outdoors, and increased social support, which could all have the beneficial effect of lowering the rate of preterm birth [34].

As may be observed in Figure 2, the morbidity of patients in the first wave was associated with an advanced age, overweight or obesity, the presence of pneumonia, the need for treatment, analytical abnormalities such as lymphopenia and thrombocytopenia, and the need for neonatal NICU along with perinatal mortality. Moreover, in the first wave, a caesarean delivery was the factor found to most contribute to perinatal morbidity (Figure 3), probably because of its close relationship with premature birth. These findings are consistent with the results of several systematic reviews [21,26] and large case series [19,20] which have examined the repercussions of COVID-19 on maternal and perinatal morbidity. During the first wave of the pandemic, rapid worsening of symptoms and the scarce therapeutic arsenal available determined the immediate termination of pregnancy [22] regardless of gestational age, and thus gave rise to more perinatal morbidity–mortality events.

In this study, we assessed maternal morbidity as a variable combining several events. One of these events, C-section showed a significant decline in the second wave and was closely related to premature birth. The differences observed between the two waves in interventionism are the outcome of improved knowledge of how to manage this infection. This determined that during the second wave, a large number of obstetric patients overcame the more severe stages of COVID-19 disease avoiding the need for their pregnancies to be terminated. Consequently, on account of the factor C-section there is much heterogeneity in this variable.

While pneumonia and the need for treatment in the mother was significantly linked to the morbidity of patients in both waves, its effect was augmented in the second wave. Moreover, pneumonia was the variable most associated with perinatal morbidity in the second wave. This increased effect of respiratory symptoms in the second wave is likely explained by both a drop in the C-section rate and preterm birth, and the increased detection of asymptomatic cases during the second wave [12], determining that patients with greater morbidity were those with respiratory symptoms and a need for treatment.

Among the strengths of our study, we should highlight the high number of pregnant women with SARS-CoV-2 infection, including 1295 participants recruited from nearly 80 centers across Spain. Our study also covers the period of unfolding of the pandemic including the first described cases among pregnant women in Spain, spanning from early March up until November 2020. Further, while fewer centers were represented in the second wave, we could still count on the participation of four out of every five hospitals at the end of this period. As far as we know, this is the first study to compare maternal, obstetric and perinatal characteristics and their impacts on maternal and perinatal morbidity in patients diagnosed during the first and second waves of the COVID-19 pandemic in Spain.

Among this study’s limitations we should stress that some response variables such as maternal and perinatal morbidity were composites of subvariables showing a different behavior pattern in the two waves. Examples of these subfactors are the C-section delivery and premature birth mentioned earlier, and this overlap should be taken into consideration when interpreting our results. Further, the magnitude of the effects analyzed could be also affected by the presence of closely related variables. Moreover, information about follow-up in some variables was not available. Due to the possible reduction in the birth rate during the second wave of the COVID-19 pandemic, a selection bias of healthier patients could be present in the study, this point should be studied when we have more information about the birth rate of the second semester of 2020.

Finally, some factors such as physical distance, air pollution and stress could explain some differences in the results of the two waves; however, these variables were not collected in our study and they could not be analyzed.

Our results thus need confirming in future studies conducted in other populations.

## 5. Conclusions

Our findings suggest that Spanish obstetric patients diagnosed with COVID-19 during the first two waves of the pandemic showed a different diagnostic profile, including more asymptomatic patients during the second wave. Differences were also observed in how the expectant mothers with COVID-19 were managed. Thus, there were fewer C-sections, and maternal and perinatal morbidity events were reduced in the second wave, while the impacts of respiratory symptoms and their severity, including a greater need for maternal treatment, were greater in this latter period. Knowledge of these changing patterns as the pandemic evolves will no doubt help improve the well-being of our patients and their newborns.

## Figures and Tables

**Table 1:** Maternal characteristics, laboratory findings, obstetrics and perinatal data and maternal/neonatal mortality and morbidity.

	Number (%) of Patients Reporting Results	First Wave	Second Wave	*p*-Value
*n*, %	1295 (100)	846 (65.3)	449 (34.7)	
**Maternal characteristics**				
Age, mean, 95% CI	1284 (99.2)	33.3 (32.8–33.7)	31.7 (31.1–32.3)	<0.001
Maternal age > 35 years, *n*, %	1284 (99.2)	358 (42.6)	145 (32.7)	<0.001
Tobacco use, *n*, %	1239 (95.7)	94 (11.6)	35 (8.2)	0.054
BMI, mean, 95% CI	1119 (86.4)	26.4 (26.1–26.8)	26.8 (26.3–27.4)	0.193
Morbidities, *n*, %	1182 (91.3)	310 (40.2)	155 (37.8)	0.431
Nuliparous, *n*, %	1281 (98.9)	316 (37.9)	175 (39.1)	0.692
Symptoms at triage, *n*, %	1103 (85.2)	457 (62.4)	155 (41.8)	<0.001
Pneumonia, *n*, %	978 (75.5)	104 (16.1)	17 (5.1)	<0.001
Treatment, *n*, %	1295 (100)	656 (77.5)	354 (78.8)	0.590
**Laboratory tests**				
Haemoglobin count (x1ꝰ/L), mean, 95% CI	892 (68.9)	12.2 (11.1–13.4)	12.4 (10.6–14.2)	0.891
Haematocrito count (x1ꝰ/L), mean, 95% CI	875 (67.6)	32.5 (31.7–33.3)	31.7 (30.5–32.9)	0.262
Leukocyte count (x1ꝰ/L), mean, 95% CI	902 (69.7)	37.2 (24.7–49.7)	10.9 (6.8–15.2)	0.003
Lymphocyte count (x1ꝰ/L), mean, 95% CI	847 (65.4)	3.6 (2.5–4.6)	4.4 (3.3–5.6)	0.319
Lymphocytopenia, *n*, %	847 (65.4)	191 (35.6)	84 (27.0)	0.009
Platelet count (x1ꝰ/L), mean, 95% CI	899 (69.4)	177.8 (169.8–185.7)	186.9 (174.6–197.1)	0.242
Thrombocytopenia, *n*, %	899 (69.4)	184 (31.4)	93 (29.8)	0.634
aPTT, mean, 95% CI	641 (50.0)	25.8 (24.1–27.5)	26.4 (22.7–30.1)	0.753
**Obstetric and perinatal characteristics**				
Obstetric morbidities, *n*, %	1057 (81.6)	299 (43.5)	150 (40.5)	0.349
Gestational age at triage, mean, 95% CI	1295 (100)	33.6 (33.0–34.1)	37.3 (37.0–37.6)	<0.001
Gestational age at delivery, mean, 95% CI	1295 (100)	38.5 (38.3–38.7)	38.7 (38.4–38.9)	0.185
Birthweight, mean, 95% CI	1275 (98.5)	3357.2 (3115.6–3198.9)	3232.4 (3176.9–3288.0)	0.036
**Maternal and perinatal mortality**				
Maternal mortality, *n*, %	1295 (100)	2 (0.2)	0 (0.0)	0.547
Perinatal mortality, *n*, %	1295 (100)	9 (1.1)	7 (1.6)	0.439
**Overall maternal morbidity**	1020 (78.8)	305 (42.4)	122 (41.7)	0.617
**COVID-19 maternal morbidity**	1295 (100)	77 (9.1)	16 (3.6)	<0.001
Oxygen therapy, *n*, %	1295 (100)	60 (7.1)	13 (2.9)	0.001
Mechanical ventilation, *n*, %	1295 (100)	15 (1.8)	2 (0.5)	0.029
Admission to ICU, *n*, %	1295 (100)	25 (3.0)	11 (2.5)	0.595
**Non COVID-19 maternal morbidity**	1015 (78.4)	276 (38.5)	118 (39.6)	0.743
C-section, *n*, %	1293 (99.8)	254 (30.1)	108 (24.1)	0.022
Haemorrhagic disorders, *n*, %	938 (72.4)	32 (4.7)	11 (4.2)	0.711
Hypertensive disorders, *n*, %	1071 (82.7)	25 (3.4)	12 83.6)	0.878
**Perinatal morbidity**	1068 (82.5)	144 (20.4)	60 (16.6)	0.137
Preterm birth, *n*, %	1295 (100)	105 (12.4)	39 (8.7)	0.039
Neonatal near miss, *n*, %	1037 (80.1)	41 (6.9)	21 (5.9)	0.964
Gestational age < 33 weeks, *n*, %	1295 (100)	36 (4.3)	17 (3.8)	0.683
Birthweight < 1750 g, *n*, %	1275 (98.5)	24 (2.9)	10 (2.3)	0.521
Apgar 5 min < 7, *n*, %	1277 (98.6)	10 (1.2)	2 (0.5)	0.165
Admission to NICU, *n*, %	1295 (100)	93 (11.0)	41 (9.1)	0.291

**Table 2:** Maternal and perinatal morbidity. Statistically significant differences are highlighted with bold fonts; n.a.: not applicable.

	Maternal Morbidity		Perinatal Morbidity	
	First Wave	Second Wave	First Wave	Second Wave
	OR/r	OR/r	OR/r	OR/r
**Maternal characteristics**				
Age, r	0.1 (0–0.2)	0.1 (0–0.3)	0.01 (−0.1–0.1)	0 (−0.1–0.1)
Maternal age > 35 years, OR	**1.5 (1.1–2.1)**	1.6 (1–2.6)	1 (0.7–1.4)	0.8 (0.4–1.5)
Tobacco use, OR	1 (0.6–1.6)	1.7 (0.7–3.8)	1.6 (0.9–2.8)	1.6 (0.6–3.8)
BMI, r	0.2 (0.1–0.2)	0.1 (0–0.2)	0 (0–0)	0 (−0.1–0.1)
BMI ≧ 25, OR	**1.9 (1.3–2.6)**	1.3 (0.8–2.1)	1.2 (0.7–1.6)	1.1 (0.6–2.1)
Morbidities, OR	**2.2 (1.6–3)**	1.6 (1–2.6)	**1.7 (1.1–2.5)**	1.7 (0.9–3.1)
Nuliparous, OR	1.2 (0.9–1.6)	0.7 (0.4–1.1)	1.4 (1–2)	0.7 (0.4–1.2)
Symptoms at triage, OR	**2.1 (1.5–3)**	1.2 (0.7–2.1)	**1.9 (1.3–2.9)**	2 (1.1–3.7)
Pneumonia, OR	**2.9 (1.8–4.5)**	**9.4 (2.1–43.3)**	1.1 (0.7–1.9)	2.4 (0.8–7.4)
Treatment, OR	**2.1 (1.4–3.2)**	**2.8 (1.6–5.2)**	1.6 (1–2.6)	1.2 (0.6–2.6)
**Laboratory tests**				
Haemoglobin count (x1ꝰ/L), r	0 (−0.1–0)	0 (−0.1–0.2)	0.1 (−0.1–0)	−0.1 (−0.2–0)
Haematocrito coun (x1ꝰ/L), r	0 (−0.2–0)	0 (−0.1–0.2)	0.1 (−0.1–0)	0 (−0.1–0.1)
Leukocyte count (x1ꝰ/L), r	0 (−0.1–0.1)	0 (−0.1–0.2)	0.1 (0–0.2)	0 (−0.2–0.1)
Lymphocite count (x1ꝰ/L), r	0 (−0.1–0.1)	−0.1 (−0.2–0.1)	0.1 (−0.1–0)	0 (−0.12–0.1)
Lymphocytopenia, OR	**1.9 (1.3–2.8)**	1.4 (0.7–2.6)	0.9 (0.6–1.4)	0.8 (0.4–1.7)
Platelet count (x1ꝰ/L), r	−0.1 (−0.2–0)	−0.1 (−0.2–0.1)	0 (−0.1–0.1)	0 (−0.1–0.1)
Thrombocytopenia, OR	**1.8 (1.2–2.6)**	1.5 (0.8–2.7)	1.1 (0.7–1.8)	1.1 (0.5–2.2)
aPTT, r	0 (−0.1–0.1)	−0.1 (−0.2–0.1)	0 (−0.1–0.1)	0 (−0.1–0.1)
**Obstetric and perinatal characteristics**				
Obstetric morbidities, OR	1.4 (1–2)	2 (1.2–3.3)	**2.1 (1.4–3)**	**4.6 (2.3–9.1)**
Gestational age at triage, r	−0.1 (−0.2–0)	−0.1 (−0.2–0.1)	−0.1 (−0.1–0)	**−0.4 (−0.5–−0.4)**
Gestational age at delivery, r	**−0.2 (−0.3–−0.2)**	−0.15 (−0.3–0)	**−0.7 (−0.7–−0.6)**	**−0.6 (−0.7–−0.6)**
Birthweight, r	−0.1 (−0.2–−0.1)	−0.2 (−0.3–0)	**−0.5 (−0.6–−0.5)**	**−0.4 (−0.6–−0.3)**
**Maternal and perinatal mortality**				
Maternal mortality, *n*, %	n.a.	n.a.	1 (n.a.–n.a.)	1 (n.a.–n.a.)
Perinatal mortality, OR	**8.3 (1–69.4)**	0.6 (0.1–3)	n.a.	n.a.
**Overall Maternal Morbidity**	n.a.	n.a.	**3.8 (2.5–5.8)**	**3.3 (1.7–6.3)**
**COVID-19 Maternal Morbidity**	n.a.	n.a.	**3 (1.8–5.1)**	**10.4 (3.4–32.4)**
Oxygen therapy, OR	n.a.	n.a.	1.7 (0.9–3.2)	**6.6 (1.9–22.3)**
Mechanical ventilation, OR	n.a.	n.a.	**10.4 (3.2–33.8)**	1 (n.a.–n.a.)
Admission to ICU, OR	n.a.	n.a.	**11.6 (4.5–30.2)**	**52.9 (6.6–426.8)**
**Not COVID-19 Maternal Morbidity**	n.a.	n.a.	**3.9 (2.6–5.8)**	**3.4 (1.8–6.7)**
C-section, OR	n.a.	n.a.	**3.8 (2.6–5.5)**	**3.2 (1.8–5.6)**
Haemorrhagic disorders, OR	n.a.	n.a.	**3.2 (1.5–6.8)**	2.6 (0.6–10.8)
Hypertensive disorders, OR	n.a.	n.a.	**4.3 (1.7–10.6)**	**23.5 (4.9–112.7)**
**Perinatal morbidity**	**3.84 (2.5–5.8)**	**3.3 (1.7–6.3)**	n.a.	n.a.
Preterm birth, OR	**3.8 (2.4–6)**	**3.5 (1.6–7.7)**	n.a.	n.a.
Apgar 5 min < 7, *n*, %	1 (n.a.–n.a.)	1 (n.a.–n.a.)	n.a.	n.a.
Admission to NICU, OR	**5.2 (3.1–8.7)**	**3.9 (1.8–8.5)**	n.a.	n.a.

In the univariate analysis, maternal morbidity events showed a strong link to perinatal mortality events in the first wave (OR: 8.3) compared to the second wave, in which maternal morbidity was more associated with pneumonia (OR: 9.4). In both waves, intense correlation was detected between maternal morbidity and perinatal morbidity events. Other variables that were associated with maternal morbidity in the first wave were maternal age higher than 35 years (OR: 1.5), BMI higher than 25 kg/m^2^ (OR: 1.9), maternal morbidities (OR: 2.2), symptoms at triage (OR: 2.1), maternal treatments (OR: 2.1), lymphocytopenia (OR: 1.9), thrombocytopenia (OR: 1.8) and GA at delivery (r: −0.2). In the second wave, maternal treatments were also linked to maternal morbidity (OR: 2.8).

## Data Availability

The data used to support the findings of the present study are available from the corresponding author upon request.
